# All New Faces of Diatoms: Potential Source of Nanomaterials and Beyond

**DOI:** 10.3389/fmicb.2017.01239

**Published:** 2017-07-05

**Authors:** Meerambika Mishra, Ananta P. Arukha, Tufail Bashir, Dhananjay Yadav, G. B. K. S. Prasad

**Affiliations:** ^1^School of Life Sciences, Sambalpur UniversityBurla, India; ^2^Department of Infectious Diseases and Pathology, University of Florida, GainesvilleFL, United States; ^3^School of Biotechnology, Yeungnam UniversityGyeongsan, South Korea; ^4^Department of Medical Biotechnology, Yeungnam UniversityGyeongsan, South Korea; ^5^School of Biochemistry, Jiwaji UniversityGwalior, India

**Keywords:** biosensors, diatoms, drug delivery, nanomaterials, nanocomposites, diatom nanotechnology

## Abstract

Nature’s silicon marvel, the diatoms have lately astounded the scientific community with its intricate designs and lasting durability. Diatoms are a major group of phytoplanktons involved in the biogeochemical cycling of silica and are virtually inherent in every environment ranging from water to ice to soil. The usage of diatoms has proved prudently cost effective and its handling neither requires costly materials nor sophisticated instruments. Diatoms can easily be acquired from the environment, their culture requires ambient condition and does not involve any costly media or expensive instruments, besides, they can be transported in small quantities and proliferated to a desirable confluence from that scratch, thus are excellent cost effective industrial raw material. Naturally occurring diatom frustules are a source of nanomaterials. Their silica bio-shells have raised curiosity among nanotechnologists who hope that diatoms will facilitate tailoring minuscule structures which are beyond the capabilities of material scientists. Additionally, there is a colossal diversity in the dimensions of diatoms as the frustule shape differs from species to species; this provides a scope for the choice of a particular species of diatom to be tailored to an exacting requisite, thus paving the way to create desired three dimensional nanocomposites. The present article explores the use of diatoms in various arenas of science, may it be in nanotechnology, biotechnology, environmental science, biophysics or biochemistry and summarizes facets of diatom biology under one umbrella. Special emphasis has been given to biosilicification, biomineralization and use of diatoms as nanomaterials’, drug delivery vehicles, optical and immune-biosensors, filters, immunodiagnostics, aquaculture feeds, lab-on-a-chip, metabolites, and biofuels.

## Introduction

Diatoms are unicellular algae (∼1–500 mm length) belonging to Class Bacillariophyceae, division Bacillariophyta, either of order centrales or pennales owing to their morphology or habitat. These phytoplanktons are further categorized into centric diatoms (Coscinodiscophyceae), pennate diatoms (Fragilariophyceae; no raphe), and pennate diatoms (Bacillariophyceae; with raphe), they exist either as unicellular or colonies, filaments, ribbons (Fragilaria), fans (Meridion), zigzags (Tabellaria), or stellate (Asterionella). Diatoms are producers within the food chain; globally contributing to almost 25% of primary productivity (Scala and Bowler, 2001). Asexual reproduction in diatoms: cell division produces two daughter cells each inheriting one parental valve, subsequently grows another smaller valve within. Owing to this size reduction division, with every generation the size of the diatom cell reduces but upon reaching a minimal size; they invert the scenario by forming an auxospore which subsequently grows larger and then undergoes size-diminishing divisions.

Diatoms can easily be acquired from the environment and transported in small quantities and proliferated to a desirable confluence. They uptake silicon from the environment and deposit it in their cell walls forming frustules which are intricate, homogenous, regularly spaced, mesoporous, siliceous nanostructures and further allow genetic modification to tailor frustules shape and pore size according to requirement. Diatoms can incorporate desired material into their frustules enhancing their use in making hybrid biosensors, bioreactors and in biotechnology, nanomedicine, photonic devices, and microfluidics. Intact frustules can be obtained from live diatoms with minimal abrasive treatment; these nanomaterials can then be further processed according to their final goal. They have been successfully used as templates for the synthesis of advanced nanostructured bio-hybrids (Nassif and Livage, 2011). Understanding and modifying the processes of biomineralization in diatoms would further accentuate its applicability in nanotechnology.

In this review, attempt to conscientiously compile the multidisciplinary applicability of diatoms in the field of nanotechnology, and biotechnology, especially in biosensor design, drug delivery, immunodiagnostics, metabolite production has been done.

## Biosilicification of Diatoms

Nature has blessed diatoms with an innate ability to uptake silicon from the environment and deposit in their cell walls; thereby generating silica shells which pose as nanomaterials with multifaceted applicability. Silicon is absorbed from the surroundings at low concentration (<1 μM) and is actively transported across membranes, as silicic acid through silicic acid transporters (SITs), leading to an internal soluble silicon pool, which subsequently makes insoluble silicon for incorporation into cell walls (Martin-Jézéquel et al., 2000; Knight et al., 2016). The biogenic silica for forming frustules is manufactured intracellularly by the polymerization of silicic acid monomers. Comparatively, low molecular weight amorphous silica is transported to the edge of Silica Deposition Vesicle (SDV) by silica-transport vesicles (STVs). Upon release into interior of the SDV, these particles diffuse till they come across the part of the breeding aggregate, unto which they stick. The surface consists of silanol groups [Si (OH)_2_ or Si–OH], facilitating them to disseminate over the surface of aggregate in a pH and temperature dependent process called ‘sintering.’ Relocation permits the molecules to restructure themselves to attain a thermodynamic stability, typically resulting in a smoothening of the aggregate surface. Silica structure formation in diatoms is normally categorized into three distinct scales progressing from the nano to the meso and finally to the microscale (Hildebrand et al., 2006, 2007). The microscale is the overall shaping of the valve and girdle bands within the SDV through active and passive molding and involves cytoskeleton, actin, and microtubules (Round et al., 1990; Van De Meene and Pickett-Heaps, 2002; Tesson and Hildebrand, 2010a; Knight et al., 2016). The organic components required for biosilica polymerization (Kröger and Wetherbee, 2000) are LCPAs (long-chain polyamines, a component of biosilica) and silaffins (Kröger et al., 2002; Poulsen and Kröger, 2004; Tesson and Hildebrand, 2010b).

**FIGURE 1:**
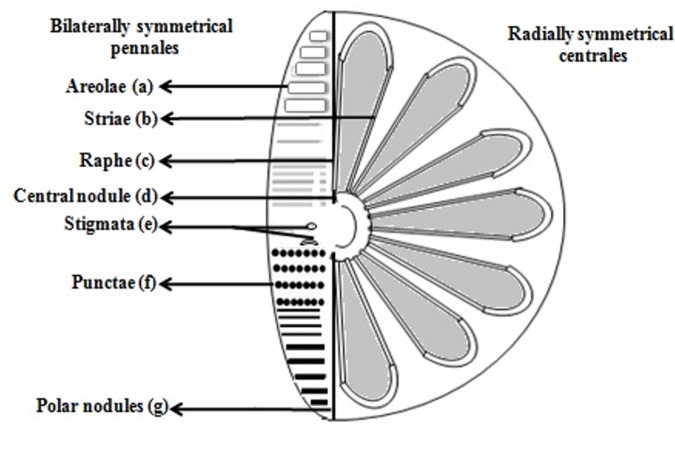
The intricate structures of the diatom. Diatom encompasses (a). Areolae (hexagonal or polygonal boxlike perforation with a sieve present on the surface of diatom, b). Striae (pores, punctae, spots or dots in a line on the surface, c). Raphe (slit in the valves, d). Central nodule (thickening of wall at the midpoint of raphe, e). Stigmata (holes through valve surface which looks rounded externally but with a slit like internal, f). Punctae (spots or small perforations on the surface, g). Polar nodules (thickening of wall at the distal ends of the raphe) diagram modified from Taylor et al. (2007).

High variability in shell shape from sparse skeletons of criss-crossing bars to barrels, pods, stars, triangles, and elaborate disks that look like flying saucers is evident. During replication, the two diatom halves (epitheca and hypotheca) and girdle bands separate and new ones are synthesized intracellularly inside the SDVs. Girdle bands may be split rings or continuous, encircling the cell or scale-like (Round et al., 1990; Hildebrand et al., 2009). Although the girdle bands are less ornate than valves they still have a structure that appears to be species dependent and are synthesized within SDV (Kröger and Wetherbee, 2000). In centric diatoms (**Figure 1**), initial valve formation occurs by the deposition of linear ribs that radiate out from the center (Round et al., 1990; Taylor et al., 2007; Hildebrand et al., 2009). Although, the basic ribbed structure of centric diatoms appears to be conserved, that being a reasonably flat ribbed structure radiating out from the center, there are variations in the nanoscale structure.

## Multipurpose Uses of Diatoms

Both live diatoms and their modified frustules have innumerable uses. Diatoms have evolved by secondary endocytobiosis, possessing atypical cell biology and genetic makeup. Advances in molecular biology and genetic engineering will unravel usage of diatoms in nanotechnology and biotechnology (Kroth, 2007). In nature, they potently remove carbon-dioxide from the atmosphere and are largely used for environmental reconstruction and audit, forensic investigation of drowning victims and water quality monitoring. The various properties leading to the use of diatoms and their frustules in different areas of technology has been summarized in **Table 1**.

**Table 1:** Properties of diatoms which make them suitable for various uses.

Uses	Property	References
Nanotechnology and material science	• Cell wall of pectin drenched with high amount of silica. • Reproducibility of the three-dimensional structures • Ability to self-replicate • Possibility of genetic engineering and low cost of production • Intricate pore sizes which can be modified • Natural variability of design includes costae (rib-like structure further longitudinal rib and axial rib), canaliculi (tube like channels), areolae (box-like), punctae (pore-like). • Heat-resistant insulation favorable for use in boilers and blast furnaces. • Very hard hence used as abrasives	Sandhage et al., 2002, 2005; Gordon and Parkinson, 2005; Hildebrand et al., 2006, 2007; Losic et al., 2006; Jeffryes et al., 2008; Mock et al., 2008; Lang et al., 2013; Rorrer and Wang, 2016

Biosensor and Forensic limnology	• Micron sized and homogenous spaced with striae • Possibility of decreasing striae width further • Prospect to cheaply create thousands of channels on a single silicon chip • Low-cost and naturally available material • Limited dispersion through ecosystems thus give identity of their environment • Frustules vary according to species and environment hence generate flora profiles for positive identification in crime scenes, drowning victims, and time of death estimation	Dempsey et al., 1997; De Stefano et al., 2009; Gordon et al., 2009; Verma, 2013

Immunoisolation, Immunodiagnostics and Immunosensors	• High sensitivity and option to chemically modify the surface to attach bioactive molecules • Filtration and encapsulation properties of diatom frustules • Probability of controlling pore size • Evades complements of the immune system	Colton, 1995; Desai et al., 1998; Townley et al., 2008; Rorrer and Wang, 2016

Filtration and water purification	• Filters micro-organisms • Homogeneous permeability and fixed pore size • Transport in small numbers • Easy multiplication post transport • Cost effective • USEPA approved	Lobo et al., 1991; Fulton, 2000

Aquaculture feed	• Lipid and amino acid rich algal content • Anti-proliferative blue green pigment • Abundantly found in nature	Duerr et al., 1998; Lebeau et al., 1999, 2000, 2002; Turpin et al., 1999

Metabolite and biofuel production, solar panel	• EPA production • Reserve food is oil, volutin, and chrysolaminarin • Production of anti-bacterial, anti-fungal, and anti-tumoral peptides • Manufacture of neutral lipids that are lipid-fuel precursors • Production of more oil under nutrient deprivation • Photosynthetic (chlorophyll a, chlorophyll c along with xanthophylls like fucoxanthin, diatoxanthin, and diadinoxanthin) and possibility of desirable engineering	Lincoln et al., 1990; Pesando, 1990; Alonso et al., 1996; Dunahay et al., 1996; Carbonnelle et al., 1998; Ramachandra et al., 2009

Bioremediation	• Heavy metal resistance due to phytochelatin synthesis or competition for metal uptake • Efficient removal of ammonium, cadmium, phosphorous, and orthophosphate • Can be re-administered to bivalves as feed • Non-invasive as are already present in the environment	Lefebvre et al., 1996; Pistocchi et al., 2000; Schmitt et al., 2001; Medarević et al., 2016

Drug delivery	• Uniform nanoscale pore structure • Chemically inert and biocompatible • Sustained release of drugs • Filtration property • Non-toxic • Species dependent drug delivery rate	Curnow et al., 2012; Zhang et al., 2013; Milovic et al., 2014; Rea et al., 2014; Vasani et al., 2015


### As a Source of Nanomaterials

Diatoms can self-replicate and can further be engineered to provide cost-effective and programmable industrialized system. Efforts to substitute silicon with metal oxides of established optical, electrical, thermal, biological, and chemical properties as germanium, titanium; even zinc have paid off bountifully (Rorrer et al., 2005; Jeffryes et al., 2008; Jaccard et al., 2009). Rorrer et al. (2005) have used diatom to controllably fabricate semiconductor titanium dioxide nanostructured by a bottom-up self-assembly course on a massively parallel scale. They metabolically inserted nano-structured TiO_2,_ forming a nano-composite of titanium and silicon in the diatom *Pinnularia* sp., by cultivating the diatom in a controlled two-stage bioreactor process. Greatly useful in dye-sensitized solar cells designed for improved light trapping efficiency and structured photocatalysts for the superior breakdown of toxic chemicals. Lang et al. (2013) have used live diatom cells to formulate organo-silica assemblies without any loss in the intricate frustule patterning. Addition of various metals to the already existant silica frustues improves their durability and usability in various nanotechnological purposes.

### As Filterant in Water Purification

Diatomaceous earth (DE) is a heterogeneous concoction of the fossil residue of dead diatoms with filtration capability. The use of diatoms over DE is advantageous because; usage of a single culture will ensure homogenous permeability and fixed pore size (Hildebrand, 2008). They can be transported cost-effectively in small numbers and cultured to desired confluence, ideal for industrial processes (Lobo et al., 1991).

### As Biodevices

Diatom cells have been grown on self-assembled monolayers. The surface of glass was activated with the addition of trifluoromethyl, methyl, carboxyl, and amino groups by the self-assembled monolayers (SAM) process following which diatom was cultured on the modified glass surface. Upon rinsing post adhesion, diatoms had formed a 2D array, thus aggrandizing their use in bio-devices development (Umemura et al., 2001). Freshwater diatoms have been used to make biosensors for water quality assessment using alternating current dielectrophoresis to chain live diatom cells in order to create a 2D array (Siebman et al., 2017).

## Industrial Applications

### Metabolite Production

Diatoms are artificially cultivated for their intracellular metabolites like eicosapentaenoic acid (EPA), essential lipids, and amino acids for pharmaceutical and cosmetic purposes (Lebeau and Robert, 2003; Hemaiswarya et al., 2011). Live diatoms as *Chaetoceros* and *Thalassiosira* species are used as larval feed (Spolaore et al., 2006), *Tetrasel missuecica*, *Thalassiosira pseudonana*, *Pavlova lutheri*, *Isochrysis galbana*, and *Skeletonema costatum* are used to feed bivalve molluscs (Hemaiswarya et al., 2011). The extracellular metabolites are used as chicken and fish feeds. *P. tricornutum* and *Nitzschia laevis* have been cultivated in various photobioreactors like perfusion cell bleeding, helical tubular photobioreactor, glass tank and glass tube outdoors photobioreactor for EPA production (Lebeau et al., 2002), used to thwart coronary heart disease, hyper-triglyceridemia, blood platelet aggregation and reduction in blood cholesterol level, preventing risk of arteriosclerosis and inflammation. EPA from more popular sources like fish oil products possess poor taste, instability and higher purification cost (Abedi and Sahari, 2014). Predominantly, *Nitzschia inconspicia* (1.9–4.7% dw EPA), *Nitzschia laevis* (2.5–2.76% dw EPA), *Navicula saprophila* and *Phaeodactylum tricornutum* (2.2–3.9% dw EPA) are cultured for EPA (Wen and Chen, 2001a,b; Lebeau and Robert, 2003; Abedi and Sahari, 2014; Wah et al., 2015). *Nitzschia inconspicia* has been reported to produce arachidonic acid around 0.6–4.7% total fatty acids (Chu et al., 1994; Lebeau and Robert, 2003). Aspartic acid and isoleucine are synthesized by *Chaetoceros calcitrans* and *S. costatum*, while leucine is synthesized only by *C. calcitrans*, ornithine by *S. costatum*, serine, glutamic acid and tyrosine by *Thalassiosira* (Derrien et al., 1998; Hildebrand et al., 2012). A strong neuroexcitatory adversary of glutamate, domoic acid is also produced by *Nitzschia navisvaringica* with about 1.7 pg cell^-1^ (Kotaki et al., 2000; Martin-Jézéquel et al., 2015). Domoic acid is also established as anti-helminthic and insecticidal (Lincoln et al., 1990; Lebeau and Robert, 2003). Antibacterial and antifungal activities of diatoms are attributed to a complex of fatty acids (Pesando, 1990; Thillairajasekar et al., 2009). *S. costatum* inhibits growth of *Vibrio* in aquaculture (Naviner et al., 1999). Organic extracts of *S. costatum* (Bergé et al., 1996) and aqueous extract of *Haslea ostrearia* (Rowland et al., 2001) are anti-tumoral, effective against human lung cancer and HIV (Hildebrand et al., 2012). A C_25_ highly branched isoprenoidpolyenes which are polyunsaturated sesterpenes oils or haslenes are responsible for anti-tumoral activities (Lebeau and Robert, 2003; Hildebrand et al., 2012).

### Biofuels

Oil as food reserve is produced by diatoms during vegetative phase which keeps them afloat while awaiting favorable conditions. Using these oils glands they also produce neutral lipids which are lipid-fuel precursors; yield a lot more oil than soybean, oil seeds and palm. Ramachandra et al. (2009) professed that diatom substantially produces more oil under stress as lesser silica or nitrogen in the culture. Micro spectrometry comparative analysis of diatom oil compared with known crude oil revealed that the former has 60–70% more saturated fatty acid than the latter. A lion’s share of the existent petrol has arisen from the fossilized diatoms. Diatoms imbibe CO_2_ and sink on the ocean floor, gets preserved to yield petroleum (Ramachandra et al., 2009; Vinayak et al., 2015).

Ramachandra et al. (2009) also established a time-saving method of producing diatom oil which reduces the production time. They have successfully modified diatom to secrete oil as contrary to storage, which facilitates daily extraction of oil. Diatoms are adhered to a solar panel on an angiosperm leaf wherein the photosynthetic diatom substitutes mesophyll. Thus stomata facilitate gaseous exchange and leaf provides a humid growth environment for diatom while it photosynthesizes. Subsequently, they have genetically engineered diatoms to directly secrete gasoline which averts additional processing (Ramachandra et al., 2009). Diatom fuels may substitute fossil fuels thus substantially reducing greenhouse gases burden. *Cyclotella cryptica* has been genetically engineered for biodiesel production (Dunahay et al., 1996). *Phaeodactylum tricornutum* Bohlin UTEX 640 was mutated to exhibit 44% higher EPA production (Alonso et al., 1996; Lebeau and Robert, 2003).

## Nanomedicine and Medical Applications

Nanomedicine employs nanomaterials, nanoelectric biosensors and molecular nanotechnology with drug delivery vehicles, diagnostic devices and physical therapy applications being equally pivotal in it. However, the major shortcoming faced by nanomedicine is toxicity, biodegradability, and environmental impact. Using diatoms or their derived frustules instead provides intricate homogeneity while also surpassing the shortcomings as they are non-toxic, biodegradable, and readily available in the environment (Bradbury, 2004; Dolatabadi and de la Guardia, 2011; Jamali et al., 2012; Li et al., 2016).

### Biosensors

The striae (**Figure 1**) in pennales are microscopic and are constantly spaced which can further be decreased using the compustat approach. The possibility of cheaply making such arrays of channels leading to Lab-on-a-chip (numerous channels on a single silicon chip) and the filtration ability of diatoms are favorable for numerous biosensor designs (Dempsey et al., 1997; Gordon et al., 2009; Siebman et al., 2017). These sensitive devices possess a biological molecular recognition constituent allied to a transducer, proficient of inducing a signal relative to the changing concentration of the molecule being sensed (Collings and Caruso, 1997). The flaw in extant biosensors is interference due to clustering of biomolecules in the circumference of the sensor. Frustules can filter; pore size is controllable, thus by incorporating a specific frustule in specific sensing chamber of biosensor, selective trafficking of the molecule can be achieved. Due to their extremely refractive nature, frustules amplify signal and thus can be used as fluorescent probe.

### Immunodiagnostics

Immunoisolating bio-encapsulation benefits from the filtration and encapsulation features of frustules. Lately, a biocapsule competent of selectively immune-isolating transplants was fashioned. The researchers used UV lithography, silicon thin film deposition and selective etching techniques (Desai et al., 1998). These capsules are adept in shielding its enclosure from defensive components of the immune system while concomitantly permitting the ample inflow of nutrients and oxygen to the transplanted tissue. Since frustules are naturally mesoporous, they are ideal vehicles for transporting nutrients to the girdled cells. In order to armor the frustules to filter immunoglobulins and complement system apparatus, the pore size is constrained in dimensions (30 nm) impenetrable to C1q and IgM (Colton, 1995). Furthermore, controlling the dimension of the pores, overall dimensions of frustule can also be altered so that hefty biocapsules adroit of enclosing several mammalian cells can be designed.

The diatom frustule can be chemically tailored for artificially tethering antibodies and bioactive molecules to it. The attached antibodies or molecules retain their inherent biological activity. These customized structures are crucial in antibody arrays and also form the basis of immunodiagnostics. As diatom biosilica requires only light and nominal nutrients hence they spawn an outstandingly low-priced and renewable starting matter (Townley et al., 2008).

### Optical Biosensors

The frustules of the central *Coscinodiscus concinnus Wm. Smith* have been chemically modified to bind to an exceedingly selective bio-probe as an antibody. Measuring the photoluminescence emission of these modified diatoms frustules, reveal the degree of antibody–ligand interaction. Diatom frustules are nanostructured, inexpensive, abundantly available naturally and also exhibit extreme sensitivity, therefore, are the ultimate entrant for the lab-on-a-chip applications (De Stefano et al., 2009).

### Drug Delivery

Homogenous pore size, constant spacing of striae, hard biosilica, genetically modifiable, chemically inert and biocompatibility are the decisive features facilitating the use of frustules as drug delivery vehicles. Pore size and rate at which the drug would be released from the diatom frustules is species-specific which gives investigators ample choices. Drug-laden diatoms can be directed to the site of release by integrating ferromagnetic elements into the frustules and then using a magnet. Currently, diatom nanotechnology is an exceedingly interdisciplinary yet a rapidly growing research front with extremely divergent applicability (Gordon and Parkinson, 2005). High-resolution imaging techniques establish a baseline for investigating biomineralization in diatoms that ultimately impact device manufacturing capabilities. Zhang et al. (2013) have efficiently used diatom for the oral delivery of drugs for gastrointestinal diseases. Usage of diatom microparticles has no toxicity rather effectively enhanced the permeability of prednisone and mesalamine while also enabling their sustained release. The use of diatom as a solid carrier for BCS Class II drugs notorious for their low water solubility for oral administration through self-emulsifying drug delivery system (SEDDS) has been reported. Two approaches using diverse self-emulsifying phospholipid suspension of carbamazepine (CBZ) first by directly mixing with diatoms, second by dispersing diatoms into its ethanolic preparation was employed. While the physical mixture procedure was more efficient, mixing with the ethanolic extract deemed faster. Both processes, however, showed prolonged longevity (Milovic et al., 2014). Diatom has also been used for transport of siRNA into tumor cells (Rea et al., 2014). Besides, diatom frustules have also been used for antibiotic delivery (Vasani et al., 2015). The genome sequences of two diatom species, *Thalassiosira pseudonana* and *Phaeodactylum tricornutum*, has already been deciphered, works on others is in progress (Armbrust et al., 2004; Bowler et al., 2008; Hildebrand et al., 2012) to effectively identify the proteins involved in fabrication of diatom skeleton features enhancing expression or direct production of desired products.

## Future Prospects

Diatoms make gargantuan variety of shapes. Some of these structures are dependent on microtubules and possibly are sensitive to microgravity. The NASA Single Loop for Cell Culture (SLCC) for culturing and observing microbes authorizes economical, low labor in-space experiments. Three diatom species were sent to the International Space Station, together with the huge (6 mm length) diatoms of Antarctica and the exclusive colonial diatom, *Bacillaria paradoxa*. The cells of *Bacillaria* moved next to each other in partial but opposite synchrony by a microfluidics method. Swift, directed evolution is achievable by using the SLCC as a compustat. Since the structural details are well conserved in hard silica, the development of normal and deviant morphogenesis can be achieved by drying the samples on a moving diatom filter paper. Owing to the massive biodiversity of diatoms, its nanotechnology will present a condensed and portable diatom nanotechnology toolkit for space exploration (Gordon and Parkinson, 2005).

Diatoms pose a novel example of a natural enigma which has been unfolded recently. There are still many unanswered questions, as the equation amid the genotype and phenotype of diatom, its further manipulation without breaking the balance of its 3D shape and pattern, methods of genetic engineering applicable. Other speculations are about the limits for diatom evolution, how can we make the most out of them and in what other fields can diatoms find use. As our comprehension of genetic composition of diatoms gets enlightened, the possibility of designing molecularly explicit architectures of large (mm) and minute (nm) dimensions would be more feasible. Genetically engineered diatoms are employed as vectors for vaccine delivery and used for enhancing the nutritional quality of the feedstuff for crustaceans and aqua-cultured fish, few diatom based vaccines have been successfully used and patented as well (Gladue and Maxey, 1994; Hempel et al., 2011; Corbeil et al., 2015; Doron et al., 2016). Various researches have been structured to find novel diatoms even in unconventional places to decipher these siliceous mysteries (Amspoker, 2016; Noga et al., 2016). The future harbors promising challenges endowed with great rewards for diatomists and nanotechnologists eventually as the research on diatoms gets more illumined.

## Author Contributions

All authors listed have made a substantial, direct and intellectual contribution to the work, and approved it for publication.

## Conflict of Interest Statement

The authors declare that the research was conducted in the absence of any commercial or financial relationships that could be construed as a potential conflict of interest.
